# SHIP-MR and Radiology: 12 Years of Whole-Body Magnetic Resonance Imaging in a Single Center

**DOI:** 10.3390/healthcare10010033

**Published:** 2021-12-24

**Authors:** Norbert Hosten, Robin Bülow, Henry Völzke, Martin Domin, Carsten Oliver Schmidt, Alexander Teumer, Till Ittermann, Matthias Nauck, Stephan Felix, Marcus Dörr, Marcello Ricardo Paulista Markus, Uwe Völker, Amro Daboul, Christian Schwahn, Birte Holtfreter, Torsten Mundt, Karl-Friedrich Krey, Stefan Kindler, Maria Mksoud, Stefanie Samietz, Reiner Biffar, Wolfgang Hoffmann, Thomas Kocher, Jean-Francois Chenot, Andreas Stahl, Frank Tost, Nele Friedrich, Stephanie Zylla, Anke Hannemann, Martin Lotze, Jens-Peter Kühn, Katrin Hegenscheid, Christian Rosenberg, Georgi Wassilew, Stefan Frenzel, Katharina Wittfeld, Hans J. Grabe, Marie-Luise Kromrey

**Affiliations:** 1 Institute of Diagnostic Radiology and Neuroradiology, University Medicine Greifswald, 17475 Greifswald, Germany; Norbert.Hosten@med.uni-greifswald.de (N.H.); Robin.Buelow@med.uni-greifswald.de (R.B.); Martin.Domin@med.uni-greifswald.de (M.D.); Katrin.hegenscheid@googlemail.com (K.H.); Christian.Rosenberg@jsd.de (C.R.); 2 Institute for Community Medicine, University Medicine Greifswald, 17475 Greifswald, Germany; voelzke@uni-greifswald.de (H.V.); CarstenOliver.Schmidt@med.uni-greifswald.de (C.O.S.); Alexander.Teumer@uni-greifswald.de (A.T.); till.ittermann@uni-greifswald.de (T.I.); Wolfgang.Hoffmann@med.uni-greifswald.de (W.H.); Jean-Francois.Chenot@med.uni-greifswald.de (J.-F.C.); 3 German Centre for Cardiovascular Research (DZHK), Partner Site Greifswald, 10785 Berlin, Germany; Matthias.Nauck@med.uni-greifswald.de (M.N.); Stephan.Felix@med.uni-greifswald.de (S.F.); Marcus.Doerr@med.uni-greifswald.de (M.D.); marcello.markus@uni-greifswald.de (M.R.P.M.); Uwe.Voelker@med.uni-greifswald.de (U.V.); Nele.Friedrich@med.uni-greifswald.de (N.F.); Stephanie.Zylla@med.uni-greifswald.de (S.Z.); Anke.Hannemann@med.uni-greifswald.de (A.H.); 4 Institute of Clinical Chemistry and Laboratory Medicine, University Medicine Greifswald, 17475 Greifswald, Germany; 5 Department of Internal Medicine B, University Medicine Greifswald, 17475 Greifswald, Germany; 6 Interfaculty Institute of Genetics and Functional Genomics, University Medicine Greifswald, 17475 Greifswald, Germany; 7 Department of Prosthetic Dentistry, Gerodontology and Biomaterials, University Medicine Greifswald, 17475 Greifswald, Germany; AmroAmer.Daboul@med.uni-greifswald.de (A.D.); Christian.Schwahn@med.uni-greifswald.de (C.S.); Torsten.Mundt@med.uni-greifswald.de (T.M.); Stefanie.Samietz@med.uni-greifswald.de (S.S.); Reiner.Biffar@med.uni-greifswald.de (R.B.); 8 Department of Restorative Dentistry, Periodontology, Endodontology, and Preventive and Pediatric Dentistry, University Medicine Greifswald, 17475 Greifswald, Germany; Birte.Holtfreter@med.uni-greifswald.de (B.H.); Thomas.Kocher@med.uni-greifswald.de (T.K.); 9 Department of Orthodontics, University Medicine Greifswald, 17475 Greifswald, Germany; Karl-Friedrich.Krey@med.uni-greifswald.de; 10 Department of Oral and Maxillofacial Surgery/Plastic Surgery, University Medicine Greifswald, 17475 Greifswald, Germany; Stefan.Kindler@med.uni-greifswald.de (S.K.); Maria.Mksoud@med.uni-greifswald.de (M.M.); 11 German Centre for Neurodegenerative Diseases (DZNE), Partner Site Rostock/Greifswald, 17489 Greifswald, Germany; 12 Clinic of Ophthalmology, University Medicine Greifswald, 17475 Greifswald, Germany; Andreas.Stahl@med.uni-greifswald.de (A.S.); Frank.Tost@med.uni-greifswald.de (F.T.); 13 Functional Imaging Unit, Institute of Diagnostic Radiology and Neuroradiology, University Medicine Greifswald, 17475 Greifswald, Germany; Martin.Lotze@med.uni-greifswald.de; 14 Institute and Policlinic of Diagnostic and Interventional Radiology, Medical University, Carl-Gustav Carus, 01307 Dresden, Germany; jens-peter.kuehn@uniklinikum-dresden.de; 15 Clinic of Orthopedics, University Medicine Greifswald, 17475 Greifswald, Germany; Georgi.Wassilew@med.uni-greifswald.de; 16 Department of Psychiatry and Psychotherapy, University Medicine Greifswald, 17475 Greifswald, Germany; Stefan.Frenzel@med.uni-greifswald.de (S.F.); Katharina.wittfeld@uni-greifswald.de (K.W.); Hans.Grabe@med.uni-greifswald.de (H.J.G.); 17 German Center of Neurodegenerative Diseases (DZNE), Rostock/Greifswald, Site Greifswald, 17489 Greifswald, Germany

**Keywords:** population-based imaging, longitudinal cohort study, whole-body magnetic resonance imaging, phenotyping, radiomics

## Abstract

The Study of Health in Pomerania (SHIP), a population-based study from a rural state in northeastern Germany with a relatively poor life expectancy, supplemented its comprehensive examination program in 2008 with whole-body MR imaging at 1.5 T (SHIP-MR). We reviewed more than 100 publications that used the SHIP-MR data and analyzed which sequences already produced fruitful scientific outputs and which manuscripts have been referenced frequently. Upon reviewing the publications about imaging sequences, those that used T1-weighted structured imaging of the brain and a gradient-echo sequence for R2* mapping obtained the highest scientific output; regarding specific body parts examined, most scientific publications focused on MR sequences involving the brain and the (upper) abdomen. We conclude that population-based MR imaging in cohort studies should define more precise goals when allocating imaging time. In addition, quality control measures might include recording the number and impact of published work, preferably on a bi-annual basis and starting 2 years after initiation of the study. Structured teaching courses may enhance the desired output in areas that appear underrepresented.

## 1. Introduction

### 1.1. History

In the 1990s, the life expectancy of the population of West Pomerania, a predominantly rural region of Germany between the Baltic Sea coast and German–Polish border, was found to be the lowest in Germany, and in an evaluation of German universities at the beginning of the 1990s, the German Council of Science and Humanities suggested that community medicine should be a research focus. Since the University of Greifswald was established 550 years ago in the West Pomeranian region, the Faculty of Medicine has built a new university hospital, constructed in two phases, in 2003 and 2012. Thus, the University of Greifswald Faculty of Medicine has a unique selling point among current German universities. The Institute for Community Medicine was established, and a deeply phenotyped population-based study on the state of health and life expectancy in the population of Western Pomerania was set up: the Study of Health in Pomerania (SHIP) project, with its three cohorts (SHIP-START, SHIP-TREND, and SHIP-NEXT), described elsewhere [1]. Of particular importance is that the study populations were selected from the entire adult population of West Pomerania [1].

The examination program was extensive, with SHIP-TREND’s baseline investigations alone comprising up to 25 h [1] including polysomnography. SHIP-START-0 had a very high response rate of 68.8% and originally included 4308 subjects. SHIP-TREND included 4420 subjects with a response rate of 50.1%. SHIP-NEXT started in 2021 and is currently performing baseline examinations.

The follow-up examinations (SHIP-START-1, -2, -3, and -4) were offered to the remaining original subjects. Since 2008, the examination programs SHIP-START-2, -3, and -4 and SHIP Trend-0 and -1 have offered whole-body MRI to all participants free of charge. Additionally, consent from subjects is requested before a contrast agent-supported examination is conducted. All consenting subjects received examinations of the heart and large vessels. Interested females received an additional contrast-enhanced MR mammography. All examinations were carried out using the same 1.5 Tesla MR scanner (Magnetom Avanato, Siemens Healthcare, Erlangen, Germany) with the same scan parameters over the whole study period in order to ensure comparability in a longitudinal setting. Carrying out the MR examinations of SHIP subjects took considerable effort. The personnel required to operate the MR scanner consists of two MRI technicians and a radiologist. In addition to educating subjects, the radiologist carried out a standardized evaluation and was present during all examinations in the control room of the MR scanner.

Twelve years after the initiation of this study, the work presented here reviews what has been achieved, similar to the ENIGMA Consortium [2,3]. An analysis of various parts of the investigation is carried out on the basis of publications in which the SHIP-MR data have been used since then, which amounts to more than 100. First, the course of the investigations is analyzed, including the treatment of incidental findings, data storage, and the pilot (feasibility) study. Next, an analysis of the scientific achievements is divided into the following sections: reference values and macroscopic anatomy; microanatomy (especially liver structure); and association studies, some of which were genome wide association studies (GWAS). Finally, we analyze which sequences included in the extensive examination program were particularly scientifically fruitful and which publications were referenced frequently. Recommendations for similar (cohort) MRI studies are then developed.

### 1.2. Different Approaches to a Population Study: Diagnostic Radiology and Epidemiology

Epidemiological follow-up studies are generally non-intervening studies. The natural course of a disease is observed retrospectively, i.e., after the disease becomes apparent clinically. A review of the influencing factors is then performed.

However, for MR examinations, non-intervention is not possible for the following reason: MRI has been used clinically since the mid-1980s. Clinicians are, therefore, experienced at diagnosing the signs of most diseases from imaging. Therefore, it seems unethical, for example, to discover and describe a mass lesion of the kidney in a population study subjecting participants to MRI without notifying the participants about such a finding, which would be diagnosed as renal cell carcinoma in clinical routine. On the other hand, a procedure in which every “non-normal” MR change is clinically clarified (biopsy or surgery after, for example, laboratory and medical history) also seemed unacceptable. Individuals may be subjected to unnecessary medical measures, which could also lead to complications (e.g., after biopsy). After discussing this matter within working groups and with the ethics committee, a clinical radiological board was assigned and tasked with determining, during the initial phase of the study, when MR findings needed to be disclosed to and clarified with SHIP subjects. In order to prevent individuals from this board from assigning patients to their own clinics, all clarifications of such “incidental findings” had to take place outside the University of Greifswald Faculty of Medicine, usually in radiological practices in the area. The findings were presented in two publications [4,5], as well as in a monograph [6,7]. However, external clarification of the findings in the SHIP-MRI was occasionally suboptimal. Some, albeit very few, findings were sometimes undetectable with less advanced equipment. This led to some delays in treatment.

Communication of the findings was extensively discussed. Disclosing incidental findings and having, above all, a waiting period before communication was fear to cause irritations in subjects. A psychologically oriented study carried out by the epidemiological side [8], however, showed that 96% of the subjects were very satisfied with the whole-body MRI procedure overall. Subjects understandably expected benefits from participating in this study. The financial equivalent of a whole-body examination in Germany is about EUR 1500 (according to the German tariff for doctors “Gebührenordnung für Ärzte, GOÄ”; see also http://www.gesetze-im-internet.de/go_1982/anlage.html Accessed date: 17 December 2021). For the physicians supervising the examination, subjects’ expectations of communicating the result of the findings were therefore quite real and had to be managed accordingly. In individual cases, concealing the results also seemed unethical. In a subject who was older and who complained of hip pain for a long time, the examination revealed abscessing inflammation of the hip joint. The subject was referred to and treated at an emergency outpatient clinic affiliated with the hospital immediately after the examination.

### 1.3. Incidental Findings

Dealing with incidental findings in scientific studies must be considered from the “therapeutic misconception” point of view [9]. This concerns the role of the “white coat”, i.e., the doctor’s status, in situations in which the doctor has to fulfill his roles as a therapist and as a scientist for the same patient [10]. An examination or intervention supervised in whatever form by a doctor incites the imperturbable expectation in patients that they will also be informed about examination results that affect their health; or, that the intervention will have some benefit for themselves. This also applies even if the subjects are explicitly told in advance that they are not to be informed about examination results in any case and must confirm their understanding of this information in writing.

In order to avoid the underlying role conflict, whole-body MRI studies similar to SHIP-MR in the Anglo-Saxon world, for example, are carried out on MR tomographs outside of hospitals and in the absence of doctors recognizable as such. They may be located in functional buildings in industrial areas and operated by personnel wearing street clothes. Moreover, participants are often reimbursed financially.

In the years up to 2008, various aspects of this situation were discussed world-wide from the legal and the ethical side and ultimately were consented to after a large, NIH-funded study [11]. At this time, incidental findings were discovered mainly in genome studies. They concerned, among other things, false paternity attributions discovered during family examinations and genetic or chromosomal variants with potentially clinical significance, even outside the actual study goal. In the past, so-called “social incidental findings” had been noticed in studies, such as bruises as result of physical abuse. With the genomic findings, there arose a legal obligation to file a complaint and to force a legal clarification. On the other hand, the legal implications in the situations described above were unclear. Works emerging from the NIH project defined the crucial questions [11,12]: Are researchers, especially non-physicians, obligated to look for incidental findings? How do researchers have to deal with incidental findings? What must be communicated to the subjects of research? How do research protocols and written consent deal with incidental findings? The study was based on a review of declarations of consent from more than 100 universities with NIH funding.

The following procedure was defined: 1. Declarations of consent must describe the risks and possible benefits for the study participants. The problem of “therapeutic misconception” (see above) had to be taken into account. A standardized procedure for dealing with incidental findings had to be initiated; 2. A procedure for incidental findings that would be found in later evaluations of stored data had to be defined; 3. A process for the detection of incidental findings had to be established; 4. The evaluation of suspected incidental findings had to be possible in a standardized way; 5. It had to be determined whether reliable incidental findings should be communicated [11].

In 2020, Richter et al. [13] examined the relationship of disclosing incidental findings (IFs) to MRI participants and subsequent biopsies and histologic examinations. Biopsies increased in participants with disclosed IFs and abnormal laboratory values after examination. Most biopsies resulted in no findings and few malignancies were diagnosed, indicating potential overtesting and overdiagnosis.

### 1.4. Data Protection and Data Sharing

Initially, the MR examinations were stored in the PACS of Greifswald University Hospital’s Institute for Radiology. For the storage, a five-digit personal identification number was assigned, which provided a pseudonymization of the examination data. From the clinical workstations, the MRI exams in the PACS could be accessed, where they appeared under the identification number. This method allowed, for example, the simple discussion of the examinations in the clinical radiological board to evaluate incidental findings, as described above. Subsequently, archiving was taken over by the Institute for Community Medicine and completely separated from clinical operations (PACS).

For the scientific use of the data, a monthly board was introduced in which data usage requests from internal and external scientists are decided. This board engages not only in the usage of MRI data, but the usage of all data collected in the context of the SHIP study, e.g., laboratory data. Unlike blood samples, MR data can be analyzed as often as desired. A full description of the data utilization board would go beyond the scope of this report (see https://www.fvcm.med.uni-greifswald.de/dd_service/data_use_intro.php, accessed on 17 December 2021). Reference should be made to corresponding publications on the SHIP project as a whole [1,4]. It is an additional function of this board to encourage cooperation of external data users with members of Greifswald University.

It is a unique advantage of the SHIP study that institutional review board certification was obtained and data privacy were solved at the beginning of the SHIP MR study for all subsequent projects.

### 1.5. Contrast Agent

Initially, it was decided to offer the subjects additional examinations with gadolinium-containing contrast agents. This decision was controversially discussed. Contrast agents were perceived as a much more invasive procedure than MRI. According to general understanding, MR imaging itself is practically free of undesirable effects, even though it exposes the patient to high-frequency energy and the constricted space of the MR tomograph’s gantry may cause negative feelings or anxiety, especially in claustrophobic people. However, both were more easily accepted by the participants than an intravenous administration of MR contrast agent. Considerations that initially led to the administration of contrast agents were the following: A study, such as the whole-body SHIP-MR study, requires considerable financial resources due to its costs. These funds are provided by the society, which in return can expect to receive reasonable results that contribute to the health of the population. In breast cancer screening and diagnostics in case of suspected breast cancer, for example, the diagnostic situation was not completely satisfactory. Although breast sonography does not utilize ionizing radiation, it only provides results at selected points of the mammary gland tissue. X-ray mammography is a very sensitive and also specific procedure because of the detection of ductal calcifications, which are present in most early breast cancers. However, ionizing radiation can, in turn, trigger breast cancer, especially in women who are sensitized due to specific mutations. Contrast agent-assisted MR mammography may be helpful in this situation. It is free of ionizing radiation and can represent the entire mammary gland parenchyma. For the whole-body MR study, an MR mammogram with contrast agent was therefore offered. This was voluntarily accepted by many study participants. All study participants were also offered a contrast agent dose for an examination of the heart and large vessels.

## 2. Methods and Material

### 2.1. Identifying Publications for This Review

Working parties represented in the Research Association Community Medicine are led by a working group leader. The names of all working group leaders were screened in the PubMed database for their authorship of publications using SHIP-MR data published between 1 January 2008 and 31 August 2021. In the research information system (FIS) of Greifswald University Medicine, publications were searched for in the same way. In addition, reviews by participants of the Community Medicine research network were searched for cited publications, which concerned topics of the review presented here. For determining the number of citations, Google Scholar was used.

### 2.2. Publications Resulting from the Examination Protocols

Publications were ranked according to the number of citations and those with more than 50 citations are presented here in a separate table (Table 1). This was intended to serve as a general impression only. Furthermore, a ranking by the quality of the publishing journal was undertaken (see Table S1) according to the (2020) Scimago Journal & Country Rank (SJR), which represents a size-independent prestige indicator that ranks journals by their ‘average prestige per article’. SJR takes the scientific influence of journals into account based on the number of citations received by a journal and the importance or prestige of the journals. Using the SJR, journals are furthermore divided into four equal quartiles. Q1 comprises the quarter of the journals with the highest values, Q2 the second highest values, Q3 the third and Q4 the lowest values. Additionally, the h-index of the publishing journals are presented.

Secondly, it was analyzed which sequences from the overall SHIP-MRI protocol resulted in published studies (Table 2). The whole protocol may be found in [4]. This approach focuses on a presentation of the topics with a view to future prioritization in other cohort studies with MRI.

## 3. Results

In the timeframe of whole-body SHIP-MRI between 2008 and until now, we could identify 105 scientific publications. Their distribution according to date of publication is displayed in Figure S1. The following section will first scrutinize the metrics of published papers, displaying the most cited and influential works. The following subheadings will give a concise overview of the published work according to research field: methodological papers (Section 3.2), epidemiological research according to anatomical region at macroscopic and microscopic level (Section 3.3.1 and Section 3.3.2), association Studies (Section 3.4), e.g., correlation analyses between imaging parameter and hormones, as well as work resulting from cooperation within international imaging consortia (Section 3.5).

### 3.1. Metrics

The most frequently cited publications are summarized in Table 1. Three of them are methodological, three are brain studies, and three are studies of the abdomen. As there is a bias towards earlier publication dates, a quality-based ranking of all published SHIP papers is presented in Table S1.

There is a certain correlation with the MR sequences leading to the most publications; the top two sequences are for anatomical brain imaging and for fat quantification. All the evaluated sequences are presented in Table 2 with regard to their resulting in individual publications. There is a wide range in use of individual sequences.

### 3.2. Published Studies of a Methodological Nature

#### 3.2.1. Pilot Study/Feasibility and Incidental Findings

**Figure 1:**
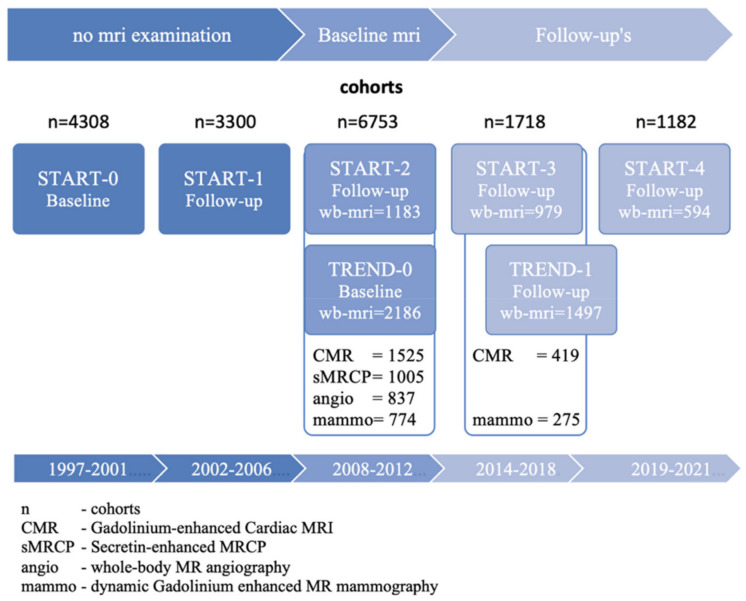
Timeline of SHIP cohorts.

The first whole-body MR study as part of SHIP-START-2 with 2333 eligible subjects was preceded by a pilot study (see Figure 1 for a timeline of this and all other SHIP-cohorts). The size of this sample was determined in advance to be 200 healthy study participants. Gender equivalence was sought. The study was meant to provide data for subjects’ willingness to participate in the study, in particular for the contrast medium-supported modules, the age distribution of participants, the practicability of a standardized report sheet, the frequency of incidental findings and the feasibility of the decisions on further clarification in a clinical radiological panel.

In total, 99 women and 101 men with a median age of 48.3 years were examined [4]. Overall, 60.4% of men and 44.4% of women agreed to participate in contrast agent-enhanced heart and breast MRI, respectively. Overall, 88% of the subjects had a total of 431 pathological findings. Of these, 89.6% were benign. In 10.4% of the incidental findings were clarified. An examination stop was necessary in five subjects due to physical complaints, in one subject due to severe claustrophobia. Then, 97% of the investigations were carried out as planned. The median examination time was 90 ± 18 min. If contrast agent-assisted modules were performed, the examination time was 143 ± 11 min (mammography and cardiac, women) or 135 ± 13 min if only the heart module was added (men). Among the findings worthy of clarification were a meningioma, an aneurysm of the internal carotids, three thyroid nodules, four adrenal gland adenomas, one mass lesion of the oropharynx, five pulmonary foci, and four prostate lesions. Contrast agent-assisted MR mammography showed three BIRADS III lesions. Additionally, found were four liver lesions, two disc prolapses in the cervical spine, four bone lesions, and one case of osteomyelitis of the lower jaw. The investigations were evaluated by two independent radiologists, the interobserver variability was good overall, not only in terms of the detection of pathological findings, but also in terms of the assessment of image quality and significant artifacts. In 2013, the random findings for the first 2500 adult study participants from SHIP-START-2 and SHIP-TREND-0 (Figure 1) were compiled again [5]. In 2 years and 7 months, 4416 SHIP study subjects were offered participation. In total, 64.6%, i.e., 2854 participants, agreed, of which 354 (8.1%) had to be excluded from MRI due to contraindications (the most important being pregnancy, pacemakers, and unclassified implants).

The sex distribution was approximately symmetrical, the median age at 53 years being slightly higher than in the pilot study. The acceptance rate of the contrast medium-based investigations was lower than in the pilot study. Overall, 1129/2500 underwent contrast-enhanced cardiac imaging, 619 men received MR cardiac imaging/angiography, and 544 women received breast imaging. Of 1330 incidental findings, 383 (36.4%) were benign, 62 (5.9%) were malignant and most were unclear (*n* = 607 and 57.7%, respectively). Nine examinations required an immediate clarification.

The panel that decided on the procedure for incidental findings was designed to define precedents. If, for example, a goiter with tracheal compression occurred once, the procedure for all future pathologies of this type was determined. As a result, the committee quickly became superfluous. For application in clinical routine, the results were compiled in a monograph [6,7].

#### 3.2.2. Normal Values/Contrast Enhancement

All study participants who participated in the MR examination were offered two additional examination modules after contrast agent injection: male and female study participants had the opportunity to have a cardiovascular examination part performed; women could additionally obtain contrast-enhanced mammography (performed first).

All subjects in SHIP-START-2, SHIP-TREND-0, and some participants of SHIP-START-3 were offered the contrast agent-supported examination modules. Then, however, the administration of contrast agents was dispensed with, as reports of an accumulation of gadolinium in the brains of recipients had been published in the meantime [109,110]. However, our own SHIP-MR study of brain accumulations provided somewhat divergent results after evaluation. For that study [55], 387 subjects were included. All participants in this subgroup had received T1-weighted images of the brain at baseline and then again five years later. The sequences and the MR scanner were identical. At baseline, 271 participants had received an intravenous dose of 0.15 mmol/kg gadobutrol. A control group of 116 study participants had not received a contrast agent. In both studies, the relative signal intensity of the thalamus, pallidum, pons, and nucleus dentatus were measured on native images. Significant differences in signal intensities were found neither between the contrast agent group and the control group at both study times nor within the contrast agent group before and after contrast agent administration. The average time between SHIP-START-2 and SHIP-START-3 was 63.6 months.

In conclusion, in an exact follow-up after a standard dose of gadobutrol no changes in signal intensities, which would have pointed to contrast agent deposition, were found. This finding is of particular importance, as other published studies usually did not have a similarly constant examination protocol with examination of subjects before and after contrast agent administration. The renunciation of the contrast agent administration may be understandable on the basis of the literature reports on gadolinium deposition. However, it weakens the SHIP-MR study in one important competitive point, since the MR examinations of the GNCS taken at the same time are also carried out without a contrast agent.

#### 3.2.3. Female Breast

The clinical use of MR mammography requires the administration of a paramagnetic contrast agent. Examinations on non-diseased patients are necessary to determine normal patterns of accumulation of the female breast under various circumstances. Only with knowledge of the normal enhancement patterns before and during menopause, under hormone replacement therapy and with different parenchyma patterns, changes in the post contrast MR mammogram can correctly be assessed. These studies were performed in the beginning of the SHIP study. Publications before SHIP were often limited to studying contrast agent passage curves of the unaffected breast in patients with unilateral breast cancer. The SHIP study, with its relatively high number of healthy women of different ages and hormone statuses, offered opportunities for knowledge gain from healthy women.

The two publications of the MR mammography working group at SHIP related to women who were examined between June 2008 and September 2011. During this period, 1475 women were included in the SHIP-MR study. Their ages ranged from 20 to 83 years. A total of 651 of them (44.1%) agreed to an additional MR mammogram. Women with allergic reactions to contrast agents or medications or breastfeeding women were not offered MR mammography. Pregnant women could not take part in SHIP-MR at all. In a first study on contrast agent kinetics of normal mammary gland tissue in dynamic MR mammography [106], 459 patients were enrolled after exclusion of women with breast implants (*n* = 12), with complete involution of the mammary gland parenchyma (*n* = 68) and with mass lesions according to BIRADS (*n* = 97). The study investigated the influence of menopausal status, oral contraceptive intake, and postmenopausal hormone therapy on contrast agent kinetics. The result showed that firstly premenopausal women had a stronger enhancement than postmenopausal women; secondly, enhancing, not circumscribed areas were more common before menopause; and thirdly hormonal therapy had only a minimal effect on the accumulation of the mammary gland parenchyma. The authors concluded that premenopausal diffuse contrast enhancements of the normal parenchyma may mark lesions and that hormone therapy does not need to be discontinued before MR mammography due to its low influence on contrast agent kinetics.

In a second study [107], 345 women from the same study period were examined with regard to contrast agent kinetics with changed inclusion and exclusion criteria. Anthropometric measures (fat content of the breasts and BMI) and the influence of menopausal status were investigated. Of the 651 women who were eligible for an MR mammogram, in addition to the above-mentioned exclusion criteria (implant, mass lesion, complete involution), ten perimenopausal women, 33 with hormone replacement therapy, and 81 study participants under oral contraceptives were excluded. The quantitative evaluation showed that both the signal intensity of the mammary gland tissue in T1-weighted images and the contrast agent enhancement varied greatly in these healthy subjects (9.3% in the lowest quintile and 47.4% in the highest). Body weight and age correlated significantly with T1 signal intensity, body weight and menopausal status with contrast agent kinetics. In women with a body weight between 50 and 100 kg, the relative contrast agent increase after 5 min was 16.6% in the group with the lowest body weight and 33.9% in women with the highest body weight.

The two studies show the social significance of contrast agent-based MR examinations in population-based studies for the population. A consideration against the individual risk from the administration of contrast agent (suspected cerebral deposits, as discussed above under “Normal Values/Contrast Enhancement”) should be carried out.

#### 3.2.4. Organ Segmentation

Automation of image analysis is obviously of great importance in studies with thousands of participants. Efforts were taken to develop tools. Gloger et al. [70] proposed a probabilistic framework that generates subject-specific probability maps for renal parenchyma tissue. Support vector machines were trained on Fourier descriptors of ground truth segmentations and used as classifiers to recognize and segment characteristic parenchyma parts. Subsequent refinements including final shape-based 3D level set segmentations led to better results than previously existing approaches. In another study, Gloger et al. [76] used manually segmented spleen masks to train support vector machines, resulting in spleen tissue priors. A 3D level set segmentation incorporates these priors for improved automated segmentation of spleen tissue of individual patients. Furthermore, Gloger et al. [74] introduced a method to automatically segment gallbladders in native and secretin-enhanced MRCP sequences. Support vector machines were trained using 2D and 3D gallbladder shape variations and an automated fuzzy c-means initial segmentation was refined by a region-based level set approach.

Ivanovska et al. [68] introduced a fast lung and trachea extraction with a three-step refinement algorithm consisting of trachea extraction, lung separation and the cavity filling of the segmented lung masks. For this purpose, K-Means and 2D/3D watershed segmentation methods were used. The group also introduced a two-step level set method for simultaneous bias field correction and tissue segmentation [25]. Due to the limitation to only two different tissue types, the application of this method excluded data such as brain MRI.

The same study group dealt with MR mammography in two studies: In the first study [105], they used a combined bias field (intensity inhomogeneity) correction and tissue segmentation, which was further refined to demarcate breast-air and breast-body boundaries. For this purpose, distance transformations and watershed segmentations were used, various hole filling methods for subsequent clean-up and a final extraction of fibro-glandular tissue being employed. Later, they introduced a two-class U-Net deep learning architecture into their previous work to improve segmentation quality [108]. As the very important bias field correction step could not be part of the segmentation step anymore, ANTs’ N4 bias field correction method [111] was now part of the new framework. Recently, Ivanovska et al. developed an approach for segmentation of structures that are relevant for the diagnosis and treatment of obstructive sleep apnea syndrome (OSAS), namely pharynx, tongue, and soft palate, from mid-sagittal MRI data [33].

Klemm et al. [36] proposed an Interactive Visual Analysis (IVA) workflow and showed its application in an exemplary way using lower back-pain cohort data. IVA provides, among other things, various tools for data selection, (3D) visualization of organ shapes and statistical measures to allow for hypothesis-driven analysis and hypothesis generation. In another study, Klemm et al. [112] improved the well-known two-dimensional correlation heat map and proposed a three-dimensional regression heat map, allowing the efficient representation of large amounts of regression models in epidemiologic studies. This allowed for improved ways of hypothesis generation.

### 3.3. Published Studies According to Anatomical Region

#### 3.3.1. Normal Morphology at Macroscopic Level

##### Brain

Hyperintense lesions of the white matter of the brain were studied in one working group [14]. The study hypothesis was that white matter hyperintensities are at least partially associated with the different types of brain atrophy seen in the elderly, but also in Alzheimer’s dementia. This association was studied in a large cohort of older subjects from the general population. The study sample included 2367 subjects, 730 from SHIP-START-2, and 1637 from SHIP-TREND-0. The median age was 52.42 years (standard deviation 13.71 years). With 1319 subjects (56.72%), the female sex is slightly predominated. Comparative parameters were available: diastolic blood pressure, HbA1c, total cholesterol, HDL and LDL, waist circumference, height, level of education, nicotine use, physical activity level, antidiabetic medication, antihypertensive medication, lipid-lowering drugs, graduated stenosis of the internal carotid artery, and the results of two tests on memory and cognitive function. Brain atrophy was quantified and typified using a machine learning technique.

It was found that a greater volume of white matter hyperintensities (WMHs) occurs in the 5th decade of life and increases beyond the age of 65. Two parameters for the early diagnosis of Alzheimer’s disease and brain atrophy were significant in the group with large volume of WMHs compared to the group with low volume of WMHs. The described increase in the volume of hyperintensities began peri-ventricularly in the age group 40 to 65 years and expanded beyond the age of 65, especially in the frontal lobes.

The results go beyond similar findings from previous studies. Due to the high number of participants in this cohort, the authors were able to identify risk factors for the development of WMHs. These are more common in smokers, and in patients with hypertension or diabetes mellitus. The cognition and memory tests showed a negative association with the total volume of WMHs. In summary, the authors suggested that risk factors for the occurrence of cardiovascular disease and WMHs of the brain substance detected in MRI may represent a “dual hit” that accelerates both the clinical manifestation and progression of neurodegenerative disease.

Voxel based morphometry (VBM) can be applied on brain MRIs of large samples since it is processed script driven (for an example see http://www.neuro.uni-jena.de/cat/ Accessed date: 17 December 2021) but needs considerable experience and time for preprocessing, quality check and statistical procedures and interpretation of data results. Since many factors are interacting on brain structure, several parameters have to be included as confounders in the analyses. Together with a need for large sample sizes, the problems of interscanner variations (multicentre studies with different scanners are problematic) offers an opportunity for utilizing SHIP-data measured on the same MRI, using the same sequence (MPRAGE), collecting data on different cohorts (e.g., START-2 and Trend-0) in a longitudinal way (e.g., START-2 and START-3). The main parameters included as confounds are demographic (age, sex see: Lotze et al. [57]), social (education time, income, social interaction), and health behavior (smoking, alcohol, sports and leisure time, BMI). Other parameters are often applied for exclusion of participants (radiologic pathology, chronic diseases, psychiatric or neurologic diseases) or for additional information of possible other impact on the brain (e.g., medication). Additional quality parameters and the total brain volume are used as confounders for the statistical analysis too. Many parameters interact in the same direction on the same areas of the brain such as chronic pain (e.g., Domin et al. [44]), smoking habits (Fritz et al. [47]), sleep deficit, or chronic stress. Some of these findings have been obtained in large samples of different research groups and seem to be quite robust. The absence of these factors increase quality of life (Hahm et al. [113]), participants show higher income (Lotze et al. [58]), and are more active in leisure sports (Eyme et al. [45]) and therefore show opposite effects on the brain’s GMV (increase in medial prefrontal GMV with higher quality of life; increase in hippocampus/amygdala GMV with higher income and less stress). In addition, all factors on the brain are dependent in their effect on brain maturation, which has to be investigated in other cohorts including younger participants. It is important to know that participants in START-2 are quite old (on average 55 years) since the cohort started as a representative cohort already 10 years before (START-0) but MRI was measured for the first time at START-2. For the TREND-0 cohort MRI was assessed initially which makes this cohort on average about 10 years younger than the START-2 participants. Therefore, GMV-effects in older age can be investigated; these are questions like a protective effect of education (Lotze et al. [58]), and sports on age related GMV decline (Eyme et al. [45]). A big challenge of SHIP-data are based in the longitudinal assessments which are however challenging to evaluate and need more elaborate statistical processes for instance for prediction of disease development on the basis of brain imaging data obtained 5 or 10 years before.

##### Eye

The eyeball shows some variance in its position within the orbit. An anterior shift in diseases such as endocrine orbitopathy is known, however additional inter-individual differences can also be assumed. In total, 1926 study participants of SHIP-MR were evaluated with regard to the position of the eyeball in the orbit [61]. MRI-measured exophthalmometry was higher in men (16.5 ± 2.2 mm) than in women (15.3 ± 2.1 mm). The axial length was 23.4 ± 0.8 mm in men and 22.8 ± 0.9 mm in women. The exophthalmometric value positively correlated with axial length and BMI. Waist circumference was also significantly correlated with the position of the bulb. The study supports the well-known fact that BMI is to be taken into account as an overarching feature in the evaluation of exophthalmometer measurements.

##### Dental, Oral, and Maxillofacial Surgery

Daboul et al. [22] examined the differences on MR images in the cross-sectional areas of the masticatory muscles in relation to age and the dental status. Their findings suggested a heterogeneous effect of age and tooth loss on the masticatory muscles. The working group also assessed the magnitude of inter-operator differences in Procrustes-based geometric morphometric analyses on craniofacial landmarks extracted from MRI, whereby an in-depth analysis of both absolute and relative error was performed [43]. They showed that inter-operator biases can be a major source of error in the analysis of large samples, as those that are becoming increasingly common in the ‘era of big data’. In another study, Daboul et al. proposed a stable landmark-based reference plane that could be used to standardize anthropometric measurements performed on population based MRI and large imaging cohorts [42].

Salti et al. [60] looked into possible associations between facial morphology, as evaluated on MRI, and attachment loss and gingival recession. Their study revealed that craniofacial morphology, specifically the cranial width and the facial index, are putative risk factors for periodontal loss of attachment.

Kindler et al. [28] studied the effect of craniofacial morphology on erupted or impacted third molars using MRI. They found that an increased maximal cranial width has a higher risk for impaction of third molars in the mandible and in the maxilla, and that individuals with a lower total anterior facial height and lower facial index also are at an increased risk for third molars impaction in the mandible. In another study [35], they looked into the association between impacted or erupted third molars and periodontal pathology, with the aim of improving the guidelines that regulate dental practitioners on the removal of the third molars. Furthermore, they investigated the systemic effect of third molars as evaluated on MR images on serum levels of inflammatory parameters and on inflammatory messenger peptide hormones in a general population sample [29]. Their study showed that participants with erupted third molars had lower levels of messenger peptide hormones, such as leptin and angiopoietin-2.

Mksoud et al. [37] examined the association between third molars position evaluated on MRI and orofacial pain. An association was found between impacted maxillary third molars and chronic orofacial pain. This will help in the decision-making process when treating patients with orofacial pain.

Liu et al. [56] developed an automated algorithm for phenotyping facial features based on landmark data obtained from 3D head MRI. They then conducted a genome-wide association study for facial shape phenotypes in multiple discovery and replication cohorts, considering almost ten thousand individuals of European descent from several countries. They found out that DNA variants in genes essential for craniofacial development contribute with relatively small effect size to the spectrum of normal variation in human facial morphology.

##### Cardiac Morphology and Function

In 1525 cardiac MR examinations normal values for left ventricular structure and functional parameters were determined and the influence of age and high blood pressure were taken into account [97]. Participants with late enhancement, high blood pressure and pre-existing cardiovascular conditions were excluded. In the reference population of 300 men and 334 women, a total of 634 participants, aged 52.1 ± 13.3 years (study population with contrast agent and cardiac MRI without exclusion criteria), the left ventricular volume decreased while the left ventricular ejection fraction increased with age. Compared to the reference population, the left ventricular end-diastolic volume was lower in men with hypertension. In both sexes, antihypertensive therapy led to a higher left ventricular ejection fraction.

The study integrated cardiac MRI with the clinical data collected in the study center of the SHIP study. From the total of 1525 SHIP participants with contrast-enhanced cardiac MRI (almost every 4th participant in the SHIP study), the information on high blood pressure, diabetes mellitus, myocardial infarction, and other cardiovascular diseases led to the formation of the reference population by exclusion. This was used to determine the left ventricular normal values. A comparable variety of medical history, clinical, ECG, and laboratory chemical information is not available in normal MRI studies.

##### Musculoskeletal

In the hip joint, the lower limit of the normal femoral neck angle is given as 120°. In an analysis of 3226 subjects, the femoral neck angle of both hip joints was measured on frontal MR images [24]. It was lower in men than in women and averaged 127° with a normal range between 114° and 140°. It became lower with age and there was a positive association with body size, BMI, and hip circumference. There was no association with body weight. The authors concluded that the previous lower limit of 120° leads to the prevalence of hip pathologies being overestimated.

Nell et al. measured normal values for the spinal canal in 2.453 subjects. Neither sex nor body weight or BMI had a significant influence on the width of the spinal canal [34].

Lumbar findings and their relationship with current and future pain was examined by Kasch et al. MRI findings were common in subjects with or without pain at baseline and showed individually or in combination no clinically relevant association with future low back pain [27].

##### Thorax

In a study of the minimal needle length required for the decompression of tension pneumothorax, Hecker et al. measured the thickness of the thoracic wall [32]. Chest wall thickness and the distance to the internal mammary artery (laceration) were measured in 2574 subjects of the SHIP study, with an average age of 53.3 years (21 to 89 years). Both sides of the thorax were used, so that 5148 values could be collected. The average thickness of the thoracic wall was 5.1 cm (standard deviation 1.4 cm). Chest wall thickness correlated with both body weight and BMI. At the entry point chosen for the measurements, the distance to the artery was 5.7 cm on the right and 5.5 cm on the left side of the body (standard deviation 0.7 cm each). The authors concluded that a needle length of 7 cm is sufficient to decompress a tension pneumothorax in the second intercostal space in the medial-clavicular line. Since the main reason for a failure of decompression of the pneumothorax is a needle that is too short, the results have direct significance for patient care.

##### Liver

MRI can be used to determine the fat content of various organs non-invasively. Hernando et al. [82] described an improved method for determining hepatic steatosis in 88 study participants who were divided into six classes according to liver fat content. (Subcutaneous adipose tissue was used as a comparison group.) The approach of the working group took into account different fat environments, which led to different fat peaks at different resonance frequencies. The correction method led to a better consideration of the spectral complexity of the fat signal and to a more accurate determination of the R2*. This approach was then used for a variety of studies using SHIP-MR data.

Kühn et al. [19] determined the fat content of the liver and iron load of liver tissue in 2561 study participants. They found a prevalence of fatty liver disease of 42.2% and iron overload of 17.4%. The combination of the two was observed in 9.3% of the subjects. Fatty liver disease was associated with increased waist circumference and, among other things, elevated blood pressure. Pietzner et al. [93] investigated the molecular mechanism that links fatty liver disease with a disrupted insulin signaling pathway. For this purpose, different metabolomics techniques were used in a group of 769 subjects with fatty liver disease and without diabetes. The role of MR here was limited to the quantification of fatty liver disease. Kromrey et al. [86] compared the accuracy of ultrasound compared to MRI as the gold standard in the diagnosis of fatty liver disease. In total, 2783 study participants with a mean BMI of 27.6 ± 4.4 kg/m^2^ participated. The inclusion criterion was participation in the SHIP-MR and the presence of an abdominal ultrasound examination carried out as part of the SHIP study. The 3D GRE sequence in breath holding technique was used for MR quantification of the fat content. PDFF and R2* maps were produced in post processing. MR showed fatty liver disease in 40% of participants (1112/2783), ultrasound in 37.8% (1052/2783). In 29.8% of the study participants, both methods diagnosed fatty liver disease. Ultrasound showed an incorrect negative result in 284 (10.2%) and false positive in 224 (8.1%), resulting in a sensitivity of 74.5% and a specificity of 86.6% for ultrasound compared to MRI. The sensitivity of ultrasound increased with the degree of fatty liver disease, the specificity was independent of fatty liver disease. The authors conclude that ultrasound of the liver can be used accordingly; the result of the ultrasound is not affected by the iron content. Naeem et al. [91] also used quantification of liver fat by transabdominal ultrasound and quantitative MRI in 1622 study subjects who had participated in an oral glucose tolerance test and in whom no type 2 diabetes mellitus was found. Study participants in whom MRI had detected fatty liver disease had a higher risk of being prediabetic or having undiagnosed type 2 diabetes mellitus. The effects were less detectable when ultrasound was used to diagnose fatty liver disease. The MR methodology is based on that described by Kühn et al. [80,86,87].

Levin et al. [90] examined the adipokines chemerin and adiponectin in their associations with liver enzymes for fatty liver disease in 3951 participants. The presence of fatty liver disease was assumed when either the ultrasound diagnosed a hyperechogenic parenchyma or the MR diagnosed an increased liver fat content. MR data were available in 1735 out of 3951 study participants. The method described by Kühn and Hernando et al. [82,89] was used. A role of adipokines in the development of fatty liver disease could be confirmed independently of inflammatory or metabolic diseases. Fischer et al. [114] published a further analysis in which MR-quantified liver fat content and visceral and subcutaneous fat were investigated in relation to various adipokines including chemerin, leptin, adiponectin, resistin, and galectin-3.

Kühn et al. [88] used liver biopsies in 95 patients and evaluated them for fat and iron content. An R2* fit with single-peak modeling led to an underestimation of the fat fraction. The same working group [89] investigated the accuracy of liver fat quantification by correlating it with spectroscopic data. Quantification with a chemical shift sequence correlated well with the results of magnetic resonance spectroscopy. However, corrections for T2* decay and other parameters were required to map the multispectral complexities of the fat. Pitchika et al. [94] investigated the importance of fatty liver disease or iron overload of the liver for associations with type 2 diabetes mellitus, metabolic syndrome, and impaired glucose metabolism. A total of 2310 study participants without proven type 2 diabetes mellitus were examined. Data on fatty liver disease and iron overload was acquired from MRI [19,88]. The importance of MR-detected fatty liver degeneration was an increased risk of type 2 diabetes mellitus, metabolic syndrome, and impaired glucose utilization in the presence of an increased ferritin concentration. Blum et al. [69] re-examined 607 study participants (SHIP-START-2) after 5 years (SHIP-START-3) for liver cysts. Methodologically, axial T2-emphasized 2D TSE images were acquired in BLADE technology. The association with liver parenchymal disease was based on R2* mapping using the previously described technique [82]. On average, 3.4 cysts were found per study participant (3.4 ± 9.0) with an average size of 13.1 ± 11.7 mm. Women were more often affected. The risk of a liver cyst increased by 2% per year of age increase. After 5 years, 24.6% of the study participants had new cysts.

##### Pancreas

Anatomical variants of the pancreatic duct were investigated by Bülow et al. [72] in 995 study participants. Under navigator triggering, T2-weighted 3D turbo spin echo MRCP images were acquired after secretin stimulation (1 U/kg body weight). Pancreas divisum was found in 9.6% of subjects, changes in the main duct in 2.4%, expansion of the lateral ducts in 16.6%, and cysts of the pancreas in 27.7%. An association with morphological signs of pancreatitis or a restriction of the function of the exocrine pancreas was not found. In another study [75], 3D MRCP was performed in 816 study participants before and after secretin stimulation (1 U/kg body weight). Only 2% showed a minor reaction to secretin (flush). Two experienced evaluators found an improvement in the visualization of the pancreatic duct at 57.4% and 58.6%, respectively. A deterioration of the visualization was found in 2.9% and 2.8%, respectively. The improvement was due, among other things, to the increase in pancreatic duct diameter. Some non-invasive quantification of exocrine pancreas function was seen in improved duodenal filling.

Kühn et al. [16] investigated the association of pancreatic fat content with type 2 diabetes and prediabetes. The proton density fat fraction (PDFF) was determined in 1367 subjects, the methodology was as described above [82]. In 740 subjects, glucose tolerance was normal, in 431 there was prediabetes and in 70 type 2 diabetes without need for medication. The pancreatic PDFF was on average 4.4% via the organ, there was no correlation with the various glucose utilization states. However, there was a positive association with age and BMI and a negative association with serum lipase activity. Kromrey et al. [85] investigated the importance of the fat load of the pancreas by correlating the PDFF with exocrine pancreatic function, defined here as the concentration of fecal elastase. The pancreatic fat content was determined in 1458 study participants. The elastase concentration was normal in 1319 subjects and decreased in 139 individuals. Study participants with decreased elastase excretion had a higher pancreatic fat content than normal elastase excreters. The authors conclude that fatty degeneration of the pancreas is a clinically significant finding.

A longitudinal study on the incidence, prevalence and pancreas-associated 5-year mortality of pancreatic cysts was conducted by Kromrey et al. [15] in 1077 study participants. Overall, 676 of them received a 5-year follow-up in the years 2014 to 2016. A mortality follow-up was conducted in 2015 for all SHIP study participants. Overall, 57.1% of the initially detected cysts showed a certain progression by size or number. A total of 12.9% showed newly occurring cysts. Pancreatic cancers were not observed. The authors concluded that pancreatic screening for cysts is not necessary. Aghdassi et al. [71] compared pancreatic volumetry with MRI and abdominal ultrasound in 342 study participants. The agreement was relatively poor, with smaller volumes resulting from ultrasound. The authors recommend taking this into account, for example, in longitudinal studies. Frost et al. [73] quantified 435 study participants with regard to duodenal fluid stimulation after secretin stimulated magnetic resonance Cholangiopancreatography (sMRCP) as part of a study on intestinal microbiota and exocrine pancreatic function. Variations in pancreatic fluid secretion measured by retention of fluid in the duodenum showed that pancreatic modulation of the intestinal microbiome is caused by acinar cell secretion.

##### Kidney

Two studies dealt with renal and perirenal changes. Mensel et al. [30] examined the prevalence and size of kidney cysts (Bosniak 1 and 2) commonly found in clinical routine in 2063 subjects (21 to 81 years old) and determined risk factors. The risk factors (former or current smokers, diabetes, BMI, systolic and diastolic blood pressure) were recorded in the SHIP study center during the physical examination and in a standardized interview. Overall, 51% of the study participants were women, the median age was 51 years; 27% of the study participants had kidney cysts, which were more common in men (34%) than women (21%). In total, 83 participants had at least one kidney cyst classified as Bosniak 2. The remaining study participants had only Bosniak 1 cysts. With increasing age, the proportion of cyst carriers increased from 14%/7% in the age group below 29 years to 55%/43% in study participants over 70 years of age (data for men/women). Men had larger cysts than women (1.5 cm versus 1.18 cm mean size), and cyst size increased with age (0.88 to 1.67 in men and 1.20 to 1.28 in women). High blood pressure (OR 1.27) and the status of the current or former smoker (OR 1.47 and 1.38) were identified as risk factors.

Another study by Mensel et al. [115] examined the prevalence of perirenal linear hyperintensities (PRH) in 1752 study participants, including 910 women and 842 men aged 21 to 81 years. These can be found on T2-weighted images of healthy subjects. Various diseases, including infections and neoplasms, are believed to be causative. The composition (urine, pus, lymphatic fluid, etc.) changes. The presence of bridging septa and an extensive lymphatic network in the perirenal space is believed to be the cause of fluid accumulation in T2-weighted image [116]. Their occurrence is considered to be associated with infection, trauma, or malignancy. The SHIP study established the fact that the prevalence of PRHs could be determined with 40.7%. Men were affected more often than women. PRHs were seen more frequently in smokers and diabetics, presumably due to venous and lymphatic stasis in the extravascular space.

#### 3.3.2. Normal Anatomy at Microscopic Level

##### Body

Kromrey et al. [87] investigated the importance of the human hemochromatosis protein (HFE) genotype for iron storage in different organs. In 483 study participants without evidence of iron storage disease, R2* levels were determined in organs that can store iron, such as the liver, spleen, pancreas, heart, bone, and lung parenchyma. Corresponding SNPs were determined for the HFR genotype. R2* values were measured in study participants without mutations and in those with at least one mutation. The reference range for R2* was determined in participants without any mutation. The mean R2* values for liver were 33.4 ± 12.7 s, spleen 24.1 ± 13.8 s, pancreas 27.2 ± 6.1 s, heart 32.7 ± 11.8 s, bone 69.3 ± 21.0 s, brain tissue 13.9 ± 1.2 s. The two groups with and without mutation in the HFE gene showed no significant difference in R2* values. The authors assessed the reference values as helpful for the diagnosis of iron storage disease. The mutations studied did not affect iron storage in tissues.

### 3.4. Association Studies

#### 3.4.1. Neuropsychiatry

With 11 publications, association studies of various neurological diseases were the most common field of study. All studies used a T1-weighted gradient echo sequence with isometric 1 mm voxels. The repetition time was 1900 ms, the echo time 3.4 ms, the flip angle 15°. Different post-processing and evaluation software were used. One publication [40], quantified white matter hyperintensities. For this purpose, an axial flair sequence in T2 weighting was used (0.9 × 0.9 mm voxel in the layer, 3 mm layer thickness, repetition time 5000 ms and echo time 325 ms, flip angle 15°). The structural part of this investigation was implemented with an MPRAGE sequence.

In terms of content, the studies dealt with the following associations: Janowitz et al. [17] investigated the association between abdominal obesity and the volume of gray matter in 758 subjects. There was a significant inverse association between waist circumference and gray matter in the brain. The relationship between alexithymia and gray matter volume was investigated by Grabe et al. [18] as the main correlate in subjects with alexithymia (*n* = 1685), resulting in a reduction in brain volume in DACC. Teipel et al. [63] investigated the association of the rs2765 single-nucleotide polymorphism (SNP of the NK3 receptor) coding gene TACR3 with the magnetic resonance imaging volume of the basal anterior lobe and hippocampus (*n* = 1967). The results suggested an association. Janova et al. [40] investigated the association between catatonic states in schizophrenic patients and genetic markers that indicate a low expression of the structural myelin protein CNP. The group recorded white matter hyperintensities in homozygous carriers compared to non-carriers and heterozygous carriers. A higher WMH volume was observed in homozygous carriers. The finding was pronounced in the frontotemporal parts of the brain and in the deep brain structures. The results were corrected intracranially for the total volume. Terock et al. [64] also investigated associations of alexithymia with cortical correlation networks (*n* = 2199). Using the Toronto Alexithymia Scale, study subjects were separated by a median split and assigned to either the high or low alexithymia network group. A significantly increased centrality in the right paracentral lobule was observed in the high alexithymia network.

Frenzel et al. [46] determined an Alzheimer’s disease score calculated from individual patterns of brain atrophy. These patterns were compared with those seen in clinical cases of Alzheimer’s disease. A total of 2154 participants of SHIP-TREND were evaluated and the score was found to be associated with performance in verbal memory tests. In addition, it distinguished well between patients and healthy controls in an independent comparison group. Markus et al. [59] investigated associations between a glucose tolerance test and the volume of gray and white matter in the brain. Fritz et al. used VBM in 315 current smokers and 659 never smokers to investigate differences in grey matter volume [47]. Grey matter was reduced in the ventromedial prefrontal cortex in smokers. Ahn et al. [39] evaluated whether intake of proton pump inhibitors (PPI) was associated with an increased risk of dementia. For this purpose, the patients’ history of PPI use was correlated with brain volumes, estimated brain age, and cognitive functions. The study examined 2653 subjects; no association between the use of PPIs and brain age was observed. Schwahn et al. [62] investigated a possible link between dental periodontal treatment and preclinical Alzheimer’s disease. Examining 177 periodontally treated subjects and 409 untreated subjects. They found that periodontal treatment had a favorable effect on Alzheimer’s disease-related brain atrophy. Wittfeld et al. [66] analyzed the relationship between cardio-respiratory fitness and global and local brain volumes. There was a positive association between gray matter volume and total brain volume with cardiorespiratory fitness. In a similar study, Jochem et al. [54] analyzed the association between physical activity and brain volume suggested by observational and interventional studies. Region of interest-based results revealed a positive association between sport activities and gray matter of the anterior cingulate cortex. However, it remained unclear whether a differential association exists between different domains of physical activity (Leisure time, Sport, and Work Index) and brain volumes. Sleep disorders seem to contribute to mental disorders as well as to neurodegeneration. Weihs et al. [65] identified an association between advanced brain aging determined by machine-learning and obstructive sleep apnea (OSA) measured by overnight polysomnography in 690 participants. The effects remained stable in the presence of various confounders (e.g., diabetes) and were partially mediated by the white blood cell count, pointing to a subclinical inflammatory process.

Genetic data in addition to MR imaging were used for association studies (GWAS). Grabe et al. [49] found that the effect on gray matter volume of several brain areas of the study participants who carried the TT genotype of the *FKBP5* gene SNP rs1360780 was moderated by childhood abuse. Interviews on childhood experiences were conducted among 1826 study participants. Experience of abuse was reported by 319 study participants. An MPRAGE sequence with isometric voxels of 1 mm^3^ was used for cortical and subcortical bilateral representations. Whole-brain voxel-based interaction analyses revealed local reductions in gray matter volume in the specified areas, which were brought about by a common effect of both childhood abuse and rs1360780 TT-carrier status. The results supported the hypothesis that the described genotype causes extensive brain changes in the described areas of the hippocampus, amygdala, cingulate cortex, and insula in the TT-carriers, if the carriers have experienced abuse in childhood. Additional examples of analyzing genetic effects on MRI-measured brain regions include two joint GWAS conducted by the Greifswald working group and a Dutch working group. The relationships between asymmetries of the two hemispheres of the brain could be investigated in larger patient populations. Guadalupe et al. [50] investigated nucleus caudatus asymmetries in a GWAS in a total of 3028 adult study participants. The study was intended to identify genetic loci that cause individual differences in the subcortical white matter and the hippocampus. The analysis used genome-wide SNP genotype data from 1276 brain imaging genetics (BIG) subjects, 932 SHIP-START and 829 SHIP-TREND subjects. The investigations were carried out on a 1.5 Tesla and a 3 Tesla system. In both studies, essentially identical MPRAGE sequences were performed with isometric 1 mm voxels. The publication contains a detailed discussion of the methodological foundations. Eventually, observed differences in hippocampal and subcortical volumes in the BIG compared to SHIP were attributed to age. The authors explicitly point to the need for the assessment of trait properties and reproducibility for such large scale studies. In a second Dutch-German GWAS MR study, Guadalupe et al. [51] investigated causes of asymmetry in and around the planum temporale in 2337 healthy subjects (technique as in the previous publication of the same working group). The planum temporale showed the strongest sex-related asymmetry of the brain regions studied. According to the authors, the results suggest steroid hormone-related genes and pathways as possible causes.

#### 3.4.2. Hormones

Other studies dealt with the influence of hormones on magnetic resonance imaging structures. In summary, the presented association studies with different hormones (thyroid, sex, lipid metabolism, stress) used isometric 3-D sequences in T1-weighting. An MPRAGE sequence was used for the representation of the brain, and a VIBE sequence for that of the abdominal visceral and subcutaneous adipose tissue. Brain imaging was realized with a head coil, body imaging used different combinations of surface coils in phased array technique. The evaluation was automated for volume determination.

Ittermann et al. [67] investigated the association of serum concentration of thyroidea-stimulating hormone TSH to the thickness of the aortic wall (AWT), the latter was used as a biomarker for atherosclerotic changes. A T1-weighted breath-hold 3D dataset was used for measurements. A high TSH value showed a significant association with increased aortic wall thickness. This was seen as an indication of a role of hyperthyroidism in aortic sclerosis. In another study, Ittermann et al. [53] used an MPRAGE sequence to map the brain to determine the hippocampal volume, as well as the volumes of white and gray matter. They found that subclinical hypothyroidism led to reduced hippocampal and white matter volumes in young subjects, while gray matter volume was not significantly altered. Hertel et al. [52] investigated the influence of oral contraceptives in 233 premenopausal women from the SHIP-TREND-0 cohort. Available were blood cortisol levels in 230 women, whole-blood transcriptome data in 226 women, and MR brain scans in 196 women. An independent cohort (SHIP-START-2) was used to replicate the results of MRI (*n* = 150 premenopausal women) and methylation analyses (*n* = 303 premenopausal women). The MR data consisted of the T1-weighted axial sequences. Analyses of the different omics data showed that oral contraceptives are associated with biological characteristics analogous to chronic psychological stressors. From MRI, the volume of the hippocampus (averaged over both hemispheres of the brain) was observed to be reduced in women using oral contraceptives.

Seyfart et al. [79] investigated the association of sex hormones with anthropometric image markers in men and women, among others. A total of 957 subjects were included. MRI was used for volumetry of abdominal adipose tissue. The quantification of the subcutaneous and visceral adipose tissue was automated and the T1-emphasized VIBE sequence already used by Witte et al. [31]. Levels of total testosterone in men were associated with decreasing BMI, decreasing hip circumference, and decreased subcutaneous adipose tissue in men. In women, both testosterone and estrone were positively associated with BMI. Leptin was inversely associated with hormone levels in men and positive in women. The study thus confirmed previous studies that found an association between sex hormones and various anthropometric markers of overweight and obesity. Genske et al. [81] investigated the association of insulin resistance, insulin sensitivity, and leptin, as well as vaspin in 981 and 698 subjects, respectively, with abdominal adipose tissue volume. The quantification of the subcutaneous and visceral adipose tissue was automated and the T1-emphasized VIBE sequence was used. Both subcutaneous abdominal adipose tissue and liver fat were associated with leptin and vaspin, but not with visceral adipose tissue. Furthermore, all three fat compartments showed strong associations with insulin sensitivity. A further study by Hannemann et al. [117] used MR-quantified visceral and subcutaneous fat and examined their relation to vitamin D concentration. The results confirmed former findings of inverse associations between adiposity measures and vitamin D. Zylla et al. [95] investigated the association of chemerin with the volumes of visceral and subcutaneous adipose tissue among 3986 study participants. Chemerin levels showed a positive association with visceral (stronger) and subcutaneous adipose tissue (weaker). An association could not be proven. Kasza et al. [83] investigated the thickness of skin-associated adipose tissue (SAT) from a whole-body 3D GRE. The method of Kühn et al. [19] was used. The thickness of the SAT showed a very high inter-individual difference. It was 1.6 times higher in female than male study participants and not associated with other signs of obesity. An animal experiment part of the publication was able to show that comparable fatty tissue deposits could be used in mice for temperature maintenance, similar to how brown adipose tissue makes this possible.

#### 3.4.3. Cardiovascular

Lorbeer et al. [102] investigated the association of cardiovascular risk factors and aortic wall thickness in 1176 study participants. Exclusion criteria were stroke or heart attack in the anamnesis. It was found that aortic wall thickness was associated with commonly known risk factors for cardiovascular disease (male sex, older age, smoking, high BMI, and increased triglyceride levels).

Based on data from 1165 participants (539 women; 46.3%) aged 21–81 years from SHIP-START-2 and SHIP-TREND-0 with both data on cardiac MRI and cardiopulmonary exercise testing, an association between lower cardiorespiratory fitness with smaller heart chambers, as well as lower values of left ventricular wall-thickness and mass, LV and LA stroke volume and cardiac output could be shown by Markus et al. [101]. In line with this, lower cardiorespiratory fitness was also related to parameters of the right ventricle (i.e., smaller chamber size, lower systolic function, stroke volume and cardiac output) in a subgroup of 941 participants with cardiac MRI (Drzyzga et al. [98]). Similar findings were reported for associations with hand grip strength (HGS), a marker of muscular fitness. Thus, lower muscular fitness was related with lower Left ventricular wall thickness and mass, as well as with smaller chamber sizes, stroke volume and cardiac output of the left ventricle, left atrium, and right ventricle. Moreover, HGS was inversely related to left ventricular diastolic stiffness and NT-proBNP values [100]. The findings of these three analyses might demonstrate the effects of an aging-related decrease in physical activity and lower muscular fitness on the heart.

Associations of glycemic parameters with glucose tolerance categories, were analyzed for associations with cardiac and arterial parameters in 1001 individuals without known diabetes (453 women, 45.3%) from SHIP-TREND-0 and KORA FF4 Study. It could be shown that higher glucose levels in the prediabetic range and insulin resistance might lead to both higher arterial stiffness and concentric remodeling of the heart [99].

#### 3.4.4. Abdomen

Two metabolomics studies used MR imaging. Pietzner et al. [93] investigated the molecular mechanism that links fatty liver disease with a disrupted insulin signaling pathway. For this purpose, different targeted and non-targeted metabolomics techniques were used in a group of 769 subjects with fatty liver disease and without diabetes. Liver fat parameters were quantified based on MR images and an associated metabolic fingerprint was identified. Interestingly a large number of new urine metabolites independent of liver injury, triglycerides, HOMA-IR, fasting glucose, or hsCRP were linked to liver fat content. Otto et al. [92] investigated the association of MR-quantified fat volumes in visceral and subcutaneous adipose tissue with a panel of mass spectrometry-derived metabolites in plasma and urine in 491 study participants. In men, an average volume of 3.35 L of visceral adipose tissue (25th percentile = 1.90; 75th percentile = 5.06) was found, in women one of 1.64 L (0.96; 2.76). Subcutaneous adipose tissue in women was more extensive at 7.02 L (5.59; 9.42) than in men at 5.17 L (3.73; 6.60). In addition to confirming findings, a positive association between visceral adipose tissue and piperines was found for the first time. MRI fat quantification was based on images taken with a body phased array coil; they were automatically identified and quantified, followed by manual correction [81].

### 3.5. Studies Resulting from Cooperation within International Imaging Consortia

Cooperation of different cohorts within large international consortia is common in the field of neurosciences and boost the power of meta- and mega-analysis. The authors of the studies presented here, those who are active in neuroscientific work, are primarily involved in the **E**nhancing **N**euro **I**maging **G**enetics through **M**eta **A**nalysis (ENIGMA) Consortium [2,61,118] and the NeuroCHARGE consortium [118]. The ENIGMA Consortium today comprises more than 1400 scientists worldwide and was founded in 2009. Today, ENIGMA has diversified into more than 50 working groups. ENIGMA has laid down standard operating procedures to harmonize the preprocessing of MRI data on its website (http://enigma.ini.usc.edu/protocols/imaging-protocols/ Accessed date: 17 December 2021). For the MR part, structured T1-weighted sequences are required in particular. (It is certainly a prerequisite for the success of this branch of neuroscience that T1-weighted MR sequences depict gray and white brain matter with good contrast in a relatively short measurement time and with good resolution.) In addition to these sequences, often of the MPRAGE type, diffusion-weighted sequences, by which the connectome is represented, and functional MR sequences such as resting-state fMRI are used.

The average number of study participants is between a few thousand [119] and GWAS studies with over fifty-thousand who were drawn from different consortia [120,121]. The MR phenotypes for these works are extracted through harmonized pipelines and are followed by quality control steps over all participating cohorts. Structural MR phenotypes (e.g., like thickness/surface measures of cortical regions or the volume of the hippocampus) are based on the cortical reconstruction and the subcortical segmentation with the freely available FreeSurfer imaging analysis suite. After this harmonized phenotype generation, associations with gender [122], age [123,124], diseases, such as depression [125,126], obesity [119], schizophrenia [127], and genome-wide associations [120,128] are conducted.

Thompson discussed possible further developments of the meta-analytic approach described above [129]. Specifically, he names the major topics of machine learning and big data, etc. Concerning MR methodology, the 7T technology, which is associated with higher spatial resolution, could be named as a possible development here (compare Steensma et al. [130], with anatomical image comparisons 3T versus 7T). The still relatively high device costs will probably prevent it from being used widely in consortia for some time. The intracranial contrasts at 7T are also influenced by factors different from those known from 1.5T and 3T. When looking through the referenced studies, which are using data from consortia, it is also noticeable that the MR tomographs, the manufacturers, the measurement sequences, firmware and software versions are often treated as commodities and merely described in attachments, sometimes those of other papers.

## 4. Discussion

### 4.1. General Timeline

Radiological methods are usually first introduced into brain imaging. New methods usually have long examination times. The brain tissue does not breathe, it has no peristalsis, and the effect of pulsations is minimal. All these effects make body applications difficult, when examination times are long. Methods that have proven themselves in brain imaging are later gradually introduced into the diagnosis of the large parenchymal organs of the body trunk. This is also true for population-based imaging where neuroimaging science came first.

A special feature of SHIP-MR is therefore the large number of examinations of the body trunk covered by the examination program. The studies on fatty liver disease, etc., were only made possible in this way. Nevertheless, in the 12 years that the SHIP-MR has been in operation, this area has been increasingly weakened: the administration of contrast medium, which enables the vascular system to be visualized, has been dispensed with. The research program thus approaches that of epidemiological and neuroscientific studies. This is partly regrettable as the influence on clinical medicine is reduced in favor of the basic sciences.

The data presented above are proof of how fruitful a whole body approach in population-based imaging can be. More than 100 publications have appeared since the start of SHIP-MR imaging. They cover a wide area of anatomic regions, of methods and of correlations.

The availability of longitudinal data from SHIP-MR is the most important advantage. In the inventory of the ENIGMA Consortium, it is explicitly pointed out that longitudinal data are missing in the relevant literature [3]. ENIGMA has delivered initial longitudinal data for neuroscience. The importance of longitudinal studies is also illustrated by the third most influential paper of the SHIP-MR study. This study by Kromrey et al. [15], on the importance of pancreatic examinations analyzes the occurrence of pancreatic carcinoma in cyst carriers. As the gold standard follow-up over a period of 5 years was set.

Longitudinal studies require unchanged examination procedures—in the presented SHIP-study the same scanner (1.5 T Magnetom Avanto, Siemens, Erlangen, Germany) was used over the whole examination period since 2008. In autumn 2021, the next round of SHIP-MRI will begin; a new equipment procurement is planned for around 2026. Among other aspects, field strength is an issue here. SHIP-MR operates at 1.5 T, the GNCS at 3.0 T. 7 T scanners are just becoming available, with studies available which compare the signal-to-noise of 7 Tesla and 3 Tesla tomographs [130]. For the prostate, for example, the authors were able to measure an increase in SNR by 1.7 to 2.8 times. The brain might profit especially from higher field strengths but longitudinal aspects are weakened.

When looking at the grouped publications presented above, the course of the successful studies can be seen on the basis of the timeline. Initially, the study design, which was the result of extensive discussions, was published [4]. Radiology-specific methodological components, i.e., the incidental findings [5] based on the ongoing work in the corresponding boards were then presented, first as a single publication and then as a German and later as an English-language monograph [6,7]. These two initial publications by Hegenscheid et al. on incidental findings and the monograph by Puls on the same topic, constitute the greatest innovations of the SHIP-MR study.

Another longitudinal study made a significant contribution to the new observations of gadolinium deposits after administration of paramagnetic contrast media [55]. The repeated MR examination 5 years after the initial examination in the SHIP-MR with administration of contrast agent was able to show that no signal intensity increases were noticeable in the follow-up examination. This does not refute, but does put the histological findings of gadolinium deposits into perspective after the administration of contrast medium.

For the processing of individual organ-specific topics, a sufficient number of subjects naturally had to be examined. Particularly successful here are two projects: on the one hand the work dealing with the MR-tomographic detection of fatty liver disease and iron overload of the liver and on the other hand the projects from the neurosciences. In this review, we have not compiled a list of automated segmentation and other evaluation procedures. However, the liver tests were segmented by hand very early on and these data were used by the segmenting scientist (J.K.) on the one hand and made available to other participants via the data management of the SHIP study on the other. In the liver tests, an important success factor was that an innovative methodological work which enabled a better T2* fit by taking into account secondary peaks of the fat signal was published relatively early on [82]. Based on this, an accurate steatosis quantification of the liver in a large number of subjects was made possible [89]. The measured degree of fatty degeneration could be validated via correlation with biopsy and spectroscopy [88], and volume determinations could be compared with other imaging modalities, such as sonography [86]. The informal working group that achieved these successes consisted of a scientist (J.K.) working at the site of the SHIP study, interested and science-savvy clinicians (Julia Mayerle, Markus M. Lerch, now Munich, Germany), and the working group of Scott Reader, Madison Wisconsin. This group had performed the actual methodological work [82]. The successful studies led to techniques of fat qualification and volumetry, as well as method comparisons being extended from the working group to other organs: Work on the pancreas, the bile duct system and later also on cyst imaging followed [72,83,88]. In a second generation, the collaboration with an associated group of Scott Reader’s working group was deepened; studies and publications of a methodological nature resulted. At this time, the fat quantifications were also used for association studies, especially those with various messenger substances from fat and glucose metabolism [25,48,64,77,79,95]. In summary, an essential condition of success was the early provision of a processed data set (segmented liver MRI), an innovative workflow that could build on then innovative sequences (multi-echo, 3D gradient echo sequence) and the collaboration with clinical colleagues.

The work in the field of neuroscience has been similarly successful: The most influential publication of the entire corpus of studies—a work from H. Grabe’s working group on periventricular medullary hyperintensities [14]—shows that studies with great social relevance (dementia, findings on routine brain MRI, etc.) are also widely referenced. Here, a different approach was chosen by utilizing an automatic segmentation of brain structures, such as gray matter, brain volume as a whole or the hippocampus, as well as changes, such as medullary hyperintensities. Here, too, a single sequence was predominantly used, the magnetization prepared rapid acquisition gradient echo sequence (MPRAGE). This provided a set of isometric voxels in T1 weighting [17,39,40,46,49,54,62,63,64,66]. Depending on the aim of the studies, different methods and software packages (e.g., FreeSurfer, SPM, FSL, or inhouse solutions) were applied to preprocess the data, extract MRI phenotypes, or conduct voxel-based morphometry analyses. The frequently cited works on the WMH additionally used a T2-emphasized FLAIR sequence.

Sequences which, according to the information in Table 2, have resulted in few or no publications nevertheless have value. In addition to the purely numerical normal ranges provided by SHIP-MR [4,5], “dormant data”, as Thompson 2020 called it [3], offer many possibilities. Whole-body DWI, e.g., can contribute to tumor staging in a way that is comparable to PET-CT. For this routine indication, descriptions of normal findings are required, which only a study such as SHIP-MR can provide to a valid degree for selected areas. Other sequences, such as the little-used heart data, may have been inadequately evaluated due to other issues. This could be remedied by publication agreements required by governance before sequences are added to the sequence protocol of a study.

The transfer of data from the SHIP-MR cohort to consortia is important. The reference section of the publication presented here has compiled to 13 publications of the ENIGMA Consortium for which SHIP data were made available and where authors from the SHIP group are named in the list of authors [2,3,119,120,121,122,123,124,125,126,127,128,129]. The overview is certainly not complete, but gives an impression on how important this use of data is.

In ENIGMA’s 10-year overview [3], Thompson points out that the data-driven approaches, as in ENIGMA and SHIP-MR, can certainly be supplemented by hypothesis-driven single center studies. For example, the 1q21.1 distal deletion and duplication copy number variant (CNV) carriers are predisposed to multiple neurodevelopmental disorders such as schizophrenia, autism, and intellectual disability. The rare cases of human carriers of this variant suffer from micro- and macrocephaly in deletion and duplication carriers, respectively. Sønderby et al. [131] used a large scale sample to precisely identify the morphological changes associated with deletion and duplication of the CNV.

Additionally, in the last few years, first works on the -omics approach have been made from SHIP data [92,93].

### 4.2. Role of Radiology in Cohort Studies

The success of the SHIP-MR study facilitated the start of a comparable nationwide cohort study, the so-called German National Cohort Study [132,133]. The National Cohort differs from the study presented here by the higher field strength of the MR tomographs used there (3.0 versus 1.5 Tesla); the higher targeted number of study participants (30,000), a modified examination spectrum with 12 organ-focused native series without contrast agent administration, the shorter measurement time (only one hour); and a central evaluation platform. The process of dealing with incidental findings [5,134] was based on the expertise gained in SHIP-MR.

The study presented here was initiated by radiology and much expertise was transferred to clinical sciences. Already, the process has gained momentum in Germany with initiation of the German National Cohort. This study aims at a tenfold increase in study subjects. Radiology as a technical specialty profits from the scientific output in the current, metrics-driven research environment. However, investigations into radiology-specific topics such as the value of contrast-enhancement are increasingly difficult to make.

In the radiologic community it is already felt that MRI as a method more and more suffers from a paucity of methodological work. Training of researchers from radiology in fields where they compete and cooperate with scientists from clinical specialties or the basic sciences is an answer, albeit not a very satisfying one. Radiology has always initiated progress with evaluation of innovations, such as image intensifiers, computed tomography, magnetic resonance tomography, and, currently, artificial intelligence. As a specialty, radiology should intensify work in methodological aspects of population imaging.

### 4.3. Conclusions

This paper aims at presenting the bigger picture of collaborative research in a population based MRI study. The research demonstrates the value of larger cohort studies using MR-imaging. Longitudinal aspects, a second focus—besides neuroimaging—on body applications and contrast agent administration proved to be of special value.

## Figures and Tables

**Table 1:** Papers from the SHIP-MR imaging study that were referenced more than 50 times.

Area of Research Referenced in (Number) Papers	UMG/External	Participant N	Relevant Publications	Main Findings (Verbal Quotations!)
Neuro, association study (237)	UMG	2367	Habes M 2016 [14]	“White matter hyperintensities also contribute independently to brain atrophy patterns in regions related to Alzheimer’s disease dementia,”
MR imaging in Population based studies, methodology (127)	UMG	194	Hegenscheid K 2009 [4]	“a large prospective, population-based study using wb-MRI is feasible and that the results of image analysis are reproducible.”
Abdomen, natural history (120)	UMG	2333	Kromrey ML 2018 [15]	“The prevalence of pancreatic cysts in the general population is unexpectedly high, and their number and size increase with age. Overall, no pancreatic cancer was observed in this collective during a 5-year follow-up.”
MR imaging in Population based studies, methodology (107)	UMG	2500	Hegenscheid K 2013 [5]	“Potentially relevant incidental findings are very common in wb-MRI research but the nature of these findings remains unclear in most cases. This requires dedicated management to protect subjects’ welfare and research integrity.”
Abdomen, epidemiology (83)	UMG	1367	Kühn JP 2015 [16]	“The presence of pancreatic fat is not related to prediabetes or diabetes, which suggests that it has little clinical relevance for an individual’s glycemic status.”
Neuro, association study (74)	UMG	2344	Janowitz D 2015 [17]	“VBM (“voxel-based morphometry”) in SHIP-2 and TREND indicated distinct associations of obesity-related factors (waist circumference and BMI) with loss of gray matter volume in mediofrontal areas.”
Neuro, association study (65)	UMG	2589	Grabe HJ 2014 [18]	“Alexiythymia was associated with areas represent(ing) language and semantic processing which might be involved in the cognitive processing of emotions and the conscious identification of feelings.”
MR imaging in Population based studies, methodology (65)	UMG	471	Schmidt CO 2013 [8]	“Despite the high satisfaction of most participants, there were numerous adverse consequences concerning the communication of incidental findings and false expectations about the likely potential benefits of whole-body-MRI.”
Abdomen, epidemiology (64)	UMG	2561	Kühn JP 2017 [19]	“In a white German population, the prevalence of fatty liver diseases and liver iron overload is 42.2% (1082 of 2561) and 17.4% (447 of 2561). Whereas liver fat is associated with predictors related to the metabolic syndrome, liver iron content is mainly associated with mean serum corpuscular hemoglobin.”

**Table 2:** The sequences used in the study program with measurement parameters and measurement time, as well as the publications in which they are mentioned in the methodology part. The MPRAGE of the brain as well as the 3D chemical shift sequence of the liver with 2D-GRE R2* mapping sequence of the liver have led to a particularly large number of publications (*n* = 20 and *n* = 17, respectively).

	Sequence	TR (ms)	TE (ms)	Flip Angle	Voxel Size	Scan Time (min)	Publication
Whole body	cor TIRM (5 stations)	4900	67	180°	1.6 × 1.6 × 5.0	12:09	Baraliakos et al., 2020 [20] Baraliakos et al., 2020 [21] Daboul et al., 2018 [22] Fischer et al., 2018 [23] Fischer et al., 2020 [24] Ivanovska et al., 2016 [25] Kasch et al., 2019 [26] Kasch et al., 2021 [27] Kindler et al., 2019 [28] Kindler et al., 2019 [29] Mensel et al., 2018 [30] Witte et al., 2017 [31] 12
Spine	sag T2 TSE (2 stations)	3760	106	180°	1.1 × 1.1 × 4.0	2:04	Baraliakos et al., 2020 [20] Baraliakos et al., 2020 [21] Hecker et al., 2016 [32] Ivanovska et al., 2021 [33] Kasch et al., 2019 [26] Kasch et al., 2021 [27] Nell et al., 2019 [34] 7
	sag T1 TSE (2 stations)	676	12	180°	1.1 × 1.1 × 4.0	2:42	Baraliakos et al., 2020 [20] Baraliakos et al., 2020 [21] Ivanovska et al., 2021 [33] Kasch et al., 2019 [26] Kasch et al., 2021 [27] Kindler et al., 2018 [35] Kindler et al., 2019 [28] Kindler et al., 2019 [29] Klemm et al., 2014 [36] Mksoud et al., 2020 [37] Nell et al., 2019 [34] 11
	sag T2*	4330	9.0/13.6/18.3/22.9/27.6	60°	1.6 × 1.6 × 5.0	1:14	0
Brain	sag T2 TSE	2610	102	180°	1.2 × 0.9 × 3.0	0:46	Chauhan et al., 2019 [38] 1
	ax T2 FLAIR	5000	325		0.9 × 0.9 × 3.0	3:47	Ahn et al., 2021 [39] Chauhan et al., 2019 [38] Habes et al., 2016 [14] Janova et al., 2018 [40] Zacharias et al., 2021 [41] 5
	ax T1 MPR	1900	3.4	15°	1.0 × 1.0 × 1.0	3:38	Ahn et al., 2021 [39] Chauhan et al., 2019 [38] Daboul et al., 2012 [42] Daboul et al., 2018 [22] Daboul et al., 2018 [43] Domin et al., 2021 [44] Eyme et al., 2019 [45] Frenzel et al., 2020 [46] Fritz et al., 2014 [47] Fritz et al., 2016 [48] Grabe et al., 2014 [18] Grabe et al., 2016 [49] Guadalupe et al., 2014 [50] Guadalupe et al., 2015 [51] Habes et al., 2016 [14] Hertel et al., 2017 [52] Ittermann et al., 2018 [53] Janova et al., 2018 [40] Janowitz et al., 2015 [17] Jochem et al., 2017 [54] Kromrey et al., 2016 [55] Liu et al., 2012 [56] Lotze et al., 2019 [57] Lotze et al., 2020 [58] Markus et al., 2017 [59] Salti et al., 2017 [60] Schmidt et al., 2019 [61] Schwahn et al., 2021 [62] Teipel et al., 2015 [63] Terock et al., 2020 [64] Weihs et al., 2021 [65] Wittfeld et al., 2020 [66] Zacharias et al., 2021 [41] 33
	ax DWI	3600	89	90°	1.2 × 1.2 × 5.0	1:10	0
	ax T2 SWI 3D	49	40	15°	1.1 × 0.9 × 3.0	2:35	0
	ax TOF angiography	23	7	25°	0.7 × 0.7 × 0.7	3:23	0
Neck	ax T1 TSE	587	11	150°	1.0 × 0.8 × 4.0	2:02	Daboul et al., 2018 [22] Kindler et al., 2018 [35] Kindler et al., 2019 [28] Kindler et al., 2019 [29] Mksoud et al., 2020 [37] 5
Chest	ax T1 VIBE	3.1	1.1	8°	1.8 × 1.8 × 3.0	0:21	Ittermann et al., 2016 [67] Ivanovska et al., 2012 [68] 2
	ax T2 HASTE	550	22	150°	2.3 × 1.8 × 5.0	0:40	Hecker et al., 2016 [32] Ivanovska et al., 2012 [68] 2
Abdomen	ax T2 FS (BLADE)	2720	116	150°	1.6 × 1.6 × 6.0	1:16	Blum et al., 2021 [69] Gloger et al., 2015 [70] Mensel et al., 2018 [30] 3
	ax T1 FLASH FS	251	4.1	70°	2.3 × 1.8 × 6.0	1:17	Aghdassi et al., 2020 [71] Mensel et al., 2018 [30] 2
	cor T2 TSE 3D (MRCP)	957	622	180°	1.0 × 1.0 × 1.5	1:42	Bülow et al., 2014 [72] Frost et al., 2019 [73] Gloger et al., 2018 [74] Kromrey et al., 2018 [15] Mensel et al., 2014 [75] Witte et al., 2017 [31] 6
	ax DWI	7160	72	90°	2.5 × 2.0 × 6.0	2:55	0
	ax T1 VIBE (4 stations)	7.5	2.4	10°	2.4 × 1.6 × 4.0	0:38	Gloger et al., 2017 [76] Mensel et al., 2016 [77] Roloff et al., 2016 [78] Seyfart et al., 2018 [79] 4
	3D three-echo-complex chemical shift (out-phase, in-phase, in-phase), multi-echo 2D-GRE including 5 in-phase TEs (R2* mapping) (WIP)	11	2.4/4.8/9.6	10°	2.24 × 1.68 × 3.0		Berg et al., 2015 [80] Blum et al., 2021 [69] Fischer et al., 2020 [24] Genske et al., 2018 [81] Hernando et al., 2013 [82] Kasza et al., 2021 [83] Kromrey et al., 2018 [84] Kromrey et al., 2019 [85] Kromrey et al., 2019 [86] Kromrey et al., 2021 [87] Kühn et al., 2012 [88] Kühn et al., 2014 [89] Kühn et al., 2015 [16] џKühn et al., 2017 [19] Levin et al., 2019 [90] Naeem et al., 2021 [91] Otto et al., 2020 [92] Pietzner et al., 2018 [93] Pitchika et al., 2021 [94] Zylla et al., 2017 [95] 20
Pelvis	ax PD TSE FS	3230	34	180°	1.6 × 1.6 × 3.0	2:43	Fischer et al., 2018 [23] Fischer et al., 2020 [24] Habes et al., 2013 [96] 3
**Heart MRI Protocol**							
	**Sequence**	**TR (mse**	**TE (ms)**	**Flip Angle**	**Voxel Size**	**Scan Time (min)**	**Publication**
Cardiac MRI pre-contrast medium	4-ChV Cine SSFP	2.7	1.1	66°	2.2 × 1.8 × 6.0	0:60	Bülow et al., 2018 [97] Drzyzga et al., 2021 [98] Markus et al., 2019 [99] Markus et al., 2021 [100] Markus et al., 2021 [101] 5
	3-ChV Cine SSFP	2.7	1.1	66°	2.2 × 1.8 × 6.0	0:10	0
	2-ChV Cine SSFP	2.7	1.1	66°	2.2 × 1.8 × 6.0	0:60	Bülow et al., 2018 [97] Drzyzga et al., 2021 [98] Markus et al., 2019 [99] Markus et al., 2021 [100] Markus et al., 2021 [101] 5
	Cardiac short- axis Cine SSFP	2.8	1.2	68°	2.0 × 1.4 × 7.0	0:54	Bülow et al., 2018 [97] Drzyzga et al., 2021 [98] Markus et al., 2019 [99] Markus et al., 2021 [100] Markus et al., 2021 [101] 5
	Cardiac axial Cine SSFP	2.8	1.2	68°	2.0 × 1.4 × 6.0	1:17	Ittermann et al., 2016 [67] Lorbeer et al., 2015 [102] Mensel et al., 2014 [103] 3
Cardiac MRI post-contrast medium	PSIR single shot	2.4	1.0	40°	3.0 × 2.1 × 6.0	0:35	Bülow et al., 2018 [97] 1
**MR Angiography Protocol for Male Subjects**							
	**Sequence**	**TR (msec)**	**TE (msec)**	**Flip Angle**	**Voxel Size**	**Scan Time (min)**	**Publication**
MR angiography pre-contrast medium	T1 FLASH 3D feet	2.5	0.9	25°	1.4 × 1.0 × 1.5	0:16	0
	T1 FLASH 3D head, abdomen, legs	2.4	0.9	25°	2.0 × 1.0 × 1.5	0:12	0
MR angiography post-contrast medium	care bolus	3354	119	30°	2.0 × 1.6 × 18.0	1:29	
	T1 FLASH 3D head, abdomen, legs	248	90	25°	2.0 × 1.0 × 1.5	0:12	Lorbeer et al., 2018 [104] 1
	T1 FLASH 3D feet	255	90	25°	1.4 × 1.0 × 1.5	0:16	0
**MR Mammography Protocol for Female Subjects**							
	**Sequence**	**TR (msec)**	**TE (msec)**	**Flip Angle**	**Voxel Size**	**Scan Time (min)**	**Publication**
MR mammography pre-contrast medium	ax TIRM	5800	56	150°	1.1 × 1.1 × 4.0	3:01	Ivanovska et al., 2014 [105] 1
	ax T2 TSE	4660	67	180°	0.9 × 0.9 × 4.0	3:17	Ivanovska et al., 2014 [105] 1
	ax DWI	7900	91	90°	1.8 × 1.8 × 4.0	4:05	Ivanovska et al., 2014 [105] 1
	3D TWIST (ax T 1 FLASH 3D)	8.9	4.5	25°	0.9 × 0.7 × 1.5		Hegenscheid et al., 2012 [106] Hegenscheid et al., 2013 [107] Ivanovska et al., 2014 [105] Ivanovska et al., 2016 [25] Ivanovska et al., 2019 [108] 5
MR mammography post-contrast medium	ax T 1 FLASH 3D (dynamic)	8.9	4.5	25°	0.9 × 0.7 × 1.5	7:03	Hegenscheid et al., 2012 [106] Hegenscheid et al., 2013 [107] Ivanovska et al., 2014 [105] 3

## Supplementary Materials

The following are available online at https://www.mdpi.com/article/10.3390/healthcare10010033/s1, Figure S1: Histogramm showing the number of SHIP publications by year, Table S1: Quality-based ranking of all published SHIP papers by Scimago Journal & Country Rank (SJR). Given is also the h-index.

## Abbreviations

BIG	Brain imaging genetics
BIRADS	Breast imaging reporting and data system
BMI	Body mass index
CNP	2′,3′-cyclic-nucleotide 3′-phosphodiesterase
CNV	Copy number variant
dACC	Dorsal anterior cingulate cortex
DWI	Diffusion-weighted imaging
FLAIR	Fluid attenuated inversion recovery
GMV	Grey matter volume
GNCS	German National Cohort Study
GWAS	Genome wide association study
HFE	Human hemochromatosis protein
Ifs	Incidental findings
MPRAGE	Magnetization prepared rapid gradient echo
NIH	National Institutes of Health
OSA	Obstructive sleep apnea
PACS	Picture archiving and communication system
PDFF	Proton density fat fraction
PPI	Proton pump inhibitors
PRH	Perirenal linear hyperintensities
R2*	Transverse relaxation rate (1/T2*)
sMRCP	Secretin stimulated magnetic resonance Cholangiopancreatography
SHIP	Study of Health in Pomerania
SHIP-MR	Magnetic Resonance Imaging in subjects from SHIP
SNP	Single-nucleotide polymorphism
T	Tesla
VBM	Voxel-based morphometry
T2*	T2 relaxation time influenced by magnetic field gradient inhomogeneities
wb-MRI	Whole-body Magnetic Resonance Imaging
WMH	White matter hyperintensities
