# Supplementary material for: SHIP-MR and Radiology: 12 Years of Whole-Body Magnetic Resonance Imaging in a Single Center
Source: Healthcare (Basel). 2021 Dec 24;10(1):33. doi: 10.3390/healthcare10010033 (PMC8775435; doi:10.3390/healthcare10010033)

healthcare

MDPI

Table S1. Quality-based ranking of all published SHIP papers by Scimago Journal & Country Rank (SJR). Given is also the h-index.

Journal	Quartil	SJR (2020)	h-index	Number of papers (n=105)
Gut	Q1	8.413	293	1
Gastroenterology	Q1	7.828	402	1
Alzheimer's and Dementia	Q1	6.713	118	1
Annals of the Rheumatic Diseases	Q1	6.333	240	2
Journal of Clinical Investigation	Q1	6.278	488	1
Brain	Q1	5.142	336	1
European Journal of Epidemiology	Q1	3.825	111	1
Plos Genetics	Q1	3.587	233	1
Journal of Clinical Periodontology	Q1	3.456	151	2
Int J Epidemiol	Q1	3.406	208	1
JAMA network open	Q1	3.278	39	1
NeuroImage	Q1	3.259	364	3
Radiology	Q1	3.118	295	4
Neurology	Q1	2.910	364	1
Neuropsychopharmacology	Q1	2.704	219	1
Cardiovascular Diabetology	Q1	2.527	75	1
Journal of the American Heart Association	Q1	2.494	85	1
Investigative Radiology	Q1	2.330	114	1
Mayo Clinic Proceedings	Q1	2.278	179	1
Sleep	Q1	2.222	207	1
Journal of Clinical Endocrinology and Metabolism	Q1	2.206	353	1
Neurobiology of Aging	Q1	2.081	186	1
Human Brain Mapping	Q1	2.005	191	3
Journal of Pain	Q1	1.972	127	2
Progress in Cardiovascular Diseases	Q1	1.929	100	2
Thyoid	Q1	1.918	142	1
Liver International	Q1	1.873	110	1
Acta Orthopaedica	Q1	1.811	141	1
Journal of Physiology	Q1	1.802	240	1
Cortex	Q1	1.786	115	1
International Journal of Obesity	Q1	1.663	225	1
Spine	Q1	1.657	254	1
European Radiology	Q1	1.606	149	9
Journal of Magnetic Resonance Imaging	Q1	1.563	160	2
Obesity	Q1	1.438	199	2
Frontiers in Psychiatry	Q1	1.363	67	1
Physics in Medicine and Biology	Q1	1.312	191	3
American Journal of Roentgenology	Q1	1.294	196	1
Journal of Trauma and Acute Care Surgery	Q1	1.250	187	1
Scientific Reports	Q1	1.240	213	6
European Thyroid Journal	Q1	1.230	10	1
Clinical Orthopaedics and Related Research	Q1	1.178	204	1
Nutrition, Metabolism and Cardiovascular Diseases	Q1	1.127	97	1
Behavioural Brain Research	Q2	1.113	171	1
Nutrition and Metabolism	Q1	1.110	84	1
Calcified Tissue International	Q1	1.078	117	1
Community Dentistry and Oral Epidemiology	Q1	1.061	101	1
Computerized Medical Imaging and Graphics	Q1	1.033	76	2
European Journal of Radiology	Q1	1.025	115	1
IEEE Transactions on Visualization and Computer Graphics	Q1	1.005	144	2

PLos ONE	Q1	0.990	332	9
Journal of Nutrition, Health and Aging	Q1	0.987	82	1
Journal of Vascular and Interventional Radiology	Q1	0.979	133	1
Endocrine Connections	Q2	0.941	23	1
European Journal of Clinical Pharmacology	Q2	0.845	108	1
Abdominal Radiology	Q2	0.824	74	3
Clinical Radiology	Q2	0.778	90	2
Journal of Oral and Maxillofacial Surgery	Q2	0.752	121	1
Neuropsychobiology	Q2	0.710	83	1
International journal of computer assisted radiology and surgery	Q1	0.701	49	2
Clinical Anatomy	Q2	0.667	71	1
Magnetic Resonance Materials in Physics, Biology and Medicine	Q2	0.585	62	1
RöFo	Q4	0.219	48	1

Figure S1. Histogramm showing the number of SHIP publications by year.

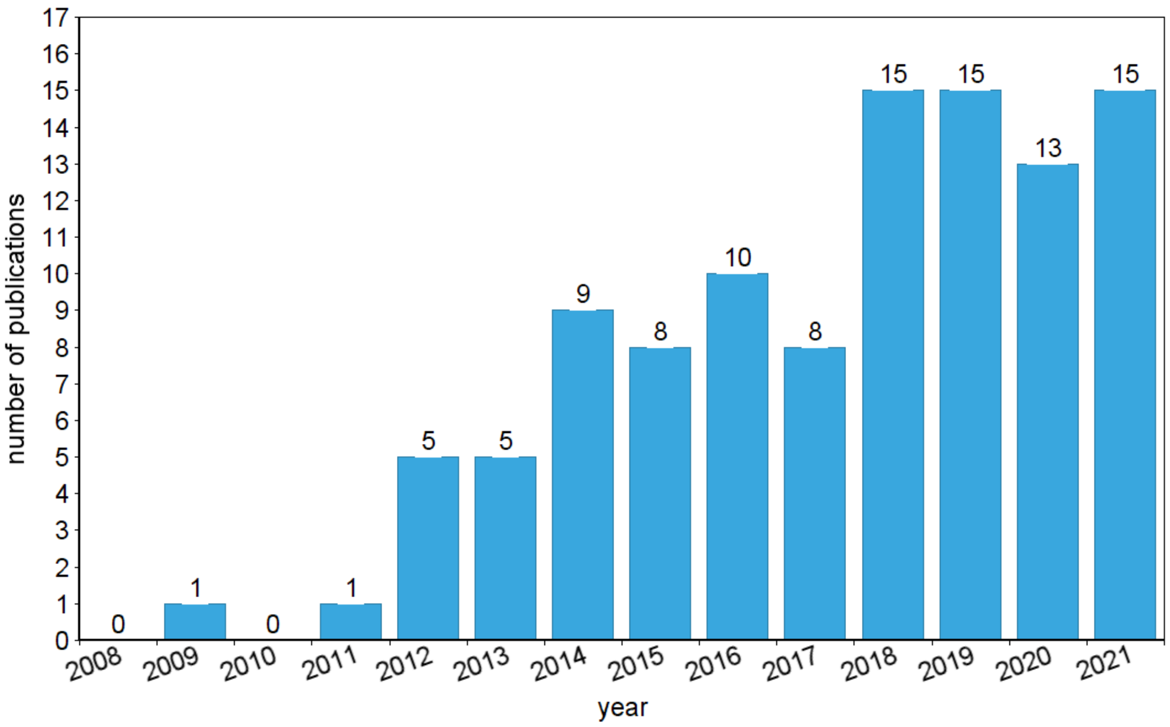

Supplement: Supplementary file 1 [file healthcare-10-00033-s001.zip › healthcare-1486881-supplementary.pdf]
